# What Is the Impact of Obesity-Related Comorbidities on the Risk of Premature Aging in Patients with Severe Obesity?: A Cross-Sectional Study

**DOI:** 10.3390/medicina61020293

**Published:** 2025-02-08

**Authors:** Alicja Dudek, Barbara Zapała, Aleksandra Gorostowicz, Ilona Kawa, Karol Ciszek, Piotr Tylec, Katarzyna Cyranka, Wojciech Sierocki, Michał Wysocki, Piotr Major

**Affiliations:** 1 2nd Department of General Surgery, Jagiellonian University Medical College, 30-688 Cracow, Poland; czerwona.chirurgia@su.krakow.pl (A.D.); ilonakawa.med@gmail.com (I.K.); ciszek.karol2@gmail.com (K.C.); tylec.piotr@gmail.com (P.T.); 2 Department of Endocrinology CMKP, Bielanski Hospital in Warsaw, 01-809 Warsaw, Poland; 3 Department of Medical Education, Jagiellonian University Medical College, 30-688 Cracow, Poland; barbara.zapala@uj.edu.pl; 4 Department of Psychiatry, Jagiellonian University Medical College, 30-688 Cracow, Polandkatarzyna.cyranka@gmail.com (K.C.); 5 Department of Metabolic Diseases, Jagiellonian University Medical College, 30-688 Cracow, Poland; 6 Department of Continuing Education, University of Oxford, Oxford OX1 2JD, UK; wojciech.sierocki@reuben.ox.ac.uk; 7 Department of General Surgery and Surgical Oncology, Ludwik Rydygier Memorial Hospital in Cracow, 31-820 Cracow, Poland

**Keywords:** obesity, premature aging, biological age markers

## Abstract

*Background and Objectives*: The relationships between aging, chronic diseases, and obesity remain complex and poorly understood. This study aimed to investigate the impact of comorbidities on premature aging in individuals with severe obesity. *Materials and Methods*: This cross-sectional study included 99 bariatric patients with severe obesity (SG) and 30 healthy volunteers (HC). SG was further divided into subgroups based on comorbidity status. Various markers of biological aging, including interleukin-6 (IL-6), C-reactive protein (CRP), telomere length (TL), attention speed, executive functions, and metabolic age, were evaluated. *Results*: Both subgroups of patients with obesity presented elevated levels of IL-6 and CRP, shorter TLs, lower outcomes in executive functioning tests, and greater metabolic age than healthy subjects. However, no significant differences were observed between patients with obesity with and without comorbidities. This study highlighted the impact of BMI on increased inflammation and revealed that hypertension and inflammation are associated with cognitive decline. *Conclusions*: These findings suggest that obesity, regardless of comorbidities, contributes to premature aging. The presence of hypertension was linked to cognitive function decline, emphasizing the multifaceted implications of obesity for the aging process.

## 1. Introduction

Increasing rates of obesity worldwide are challenging health systems. Obesity and its associated comorbidities extend beyond individual health concerns; they represent a pressing public health crisis, affecting the socioeconomic situation due to the worsening health status of young working people [1,2].

Severe obesity and aging present common features, leading to the development of chronic diseases such as atherosclerosis, cardiovascular disease, insulin resistance, type 2 diabetes, osteoarthritis, and certain types of cancer, which cumulatively lead to disability [3,4,5,6,7]. The primary mechanisms driving the aging process are attributed to a persistent, low-level inflammatory phenomenon known as ‘inflammaging’. This phenomenon is instigated by the gradual build-up of tissue-damaging proinflammatory elements, the activation of aging-promoting transcription factors (such as NFκB), compromised autophagy, impaired immune functionality, and an elevated rate of cellular aging. Underlying obesity is the accumulation of low-level inflammation, leading to the development of oxidative stress and increased ROS formation, which damages the nucleus and promotes telomere shortening and cellular senescence [8,9].

Obesity-generated comorbidities further exacerbate chronic inflammation in the body by activating T lymphocytes, promoting the expression of proinflammatory factors, and increasing apoptosis and tissue fibrosis [10,11,12]. These processes amplify the already deleterious effects of obesity on biological aging and highlight the need to disentangle the individual contributions of obesity and comorbidities to premature aging. While previous research has demonstrated the association between specific comorbidities (e.g., type 2 diabetes, hypertension) and increased inflammation, oxidative stress, and telomere attrition [13,14,15,16], it remains unclear whether the aging-related changes are determined by obesity itself or the cumulative effect of comorbidities.

Given the increasing prevalence of severe obesity, it is essential to evaluate how obesity interacts with comorbidities to accelerate biological aging and to identify whether the presence of comorbidities leads to additive or synergistic effects. Investigating differences between individuals with severe obesity who have comorbidities and those who do not is particularly relevant, as it may clarify whether interventions targeting comorbidities could mitigate aging-related outcomes in populations with severe obesity. Moreover, exploring this distinction may reveal whether the mechanisms of premature aging differ between these subgroups, thereby informing more tailored prevention or treatment strategies.

There are several types of biological age markers from which we can distinguish inflammatory levels, telomere length, cognitive abilities, and metabolic age [17]. Telomere shortening is a natural process that progresses with age, but accumulating inflammatory processes, oxidative stress, and DNA-damaging agents lead to excessive telomere attrition and shortening [18]. Upon aging, a substantial portion of the population experiences cognitive decline, which constitutes the foremost risk factor for the onset of neurodegenerative conditions, such as Alzheimer’s disease (AD). Obesity in mid-life represents a noteworthy risk factor for the emergence of AD and vascular dementia during the later stages of life; however, the factors and mechanisms underlying the deterioration of cognitive function remain unclear [19,20]. Metabolic age is a parameter referring to metabolic efficiency. It is clear that the basal metabolic rate decreases almost linearly with age; however, an increase in body fat and a decrease in muscle mass significantly impact the deterioration of metabolic age.

Given this context, the present study aimed to verify the effects of obesity per se or associated comorbidities on features of premature aging and to investigate correlations between individual obesity-related comorbidities and biological age markers. By comparing patients with severe obesity with and without comorbidities, this study provides a comprehensive assessment of the independent and combined effects of obesity and its associated conditions on biological age markers, offering new insights into the multifaceted implications of severe obesity for the aging process. The novelty of the present study is the simultaneous indication of different aspects of aging through a comprehensive assessment of various markers of biological age.

## 2. Materials and Methods

### 2.1. Study Design

This prospective cross-sectional study included 129 subjects aged between 18 and 65 years. The participants were recruited from the general surgery department of an academic hospital and a specialized private medical center dedicated to promoting well-being and extending lifespans. The participants were divided into two groups based on BMI (kg/m^2^). Individuals with severe obesity were qualified for laparoscopic sleeve gastrectomy in accordance with the guidelines of The Metabolic and Bariatric Surgery Chapter of the Association of Polish Surgeons with a body mass index (BMI) ≥ 40 kg/m^2^ or BMI ≥ 35 kg/m^2^ with comorbidities [21]. The patients with obesity were divided into two subgroups: patients with obesity and comorbidities (O+C) (n = 78) and patients without comorbidities (O−C) (n = 31). Eligibility for the groups was accomplished based on past medical history, laboratory results, and results of tests routinely conducted before bariatric surgery. The control group (n = 30) comprised participants who had never suffered from obesity or comorbidities and maintained a BMI within the range of 18.5 to 24.9 kg/m^2^.

Subjects were excluded from the study if they did not participate in the study, exhibited active inflammation, suffered from depression, neurological disorders, or any condition affecting cognitive function, consumed medications impairing cognitive function, or experienced pregnancy within the preceding 6 months.

The medical interviews, body composition, markers of inflammation, interleukin 6 (IL-6) and C-reactive protein (CRP), telomere length (TL), cognitive function tests, and metabolic age were assessed in all participants.

First, the differences in the examined parameters were compared among the three groups (O+C, O−C, and HC). Second, the relationships between BMI, comorbidities, inflammatory markers, telomere length, cognitive function, and metabolic age were assessed. The main components of metabolic syndrome—hypertension, dyslipidemia, and prediabetes/diabetes—were selected as comorbidities.

### 2.2. Endpoints of the Study

The primary endpoint was to assess the associations between markers of biological aging (inflammatory markers, telomere length, cognitive function, and metabolic age) and severe obesity, with subgroup analyses based on the presence or absence of obesity-related comorbidities.

The second endpoint explored the relationship between individual obesity-related comorbidities (prediabetes/diabetes, hypertension, and atherogenic dyslipidemia) and markers of biological aging (inflammatory markers, telomere length, cognitive function, and metabolic age).

### 2.3. Biological Age Markers

IL-6 and CRP examination

Blood sample collection and storage:

Blood samples were collected from the cephalic vein of all study participants after an overnight fast. Then, all the samples were centrifuged at 1000× *g* for 15 min at room temperature, after which the serum was collected. The serum specimens were stored at −80 °C until further analysis.

Quantification of serum IL-6 and CRP

The IL-6 and CRP concentrations in the serum were measured using commercially available enzyme-linked immunosorbent assay (ELISA) kits (Bio-Techne, Dublin, Ireland). All procedural steps were performed according to the manufacturer’s instructions and recommendations. The concentrations of IL-6 and CRP were expressed as pg/mL and mg/L, respectively.

Telomere length

Extraction of Genomic DNA:

A NucleoSpin Blood Purification Kit (Macgerey Nagel, Düren, Germany) was utilized to extract genomic DNA from blood samples following the manufacturer’s guidelines. The resulting DNA was dissolved in EB buffer. Subsequently, each DNA sample was quantified for concentration using a Qubit fluorometer (Thermo Fisher Scientific, Waltham, MA, USA), and the integrity of the DNA was assessed using an Agilent 2100 Bioanalyzer.

Telomere Length Analysis

Digital PCR technology was employed to determine the absolute telomere length (aTL) values. High-molecular-weight DNA was diluted with molecular-grade water to achieve a concentration of 1 ng/2 µL. For analysis, 1 ng of DNA was used as input material. All samples were measured in triplicate on the same plate and then averaged. PCR amplification was conducted in a Qiagen QIacuity Digital PCR Machine. The thermal profile for digital PCR was adapted based on the qPCR method developed by Cowton with specific modifications (details available upon request) [22]. Each plate included a nontemplate control, and three established reference DNA controls were run in triplicate as positive controls. The forward and reverse telomere primer sequences were adapted from Cowton and are as follows:

Forward: 5′-cggtttgtttgggttt-gggtttgggtttgggtttgggtt-3′.

Reverse: 5′-ggcttgccttacccttacccttacccttacccttaccct-3′.

Cognitive function assessment

In each patient, cognitive functions were examined. Test interpretation was conducted by a clinical psychologist affiliated with the university hospital.

We used the Color Trails Test (CTT test) to assess the speed of task solving, metastability of attention, and sequential processing (Pracownia Testów Psychologicznych PTP, Warsaw, Poland). The CTT test comprises two components, CTT-1 and CTT-2. In CTT-1, the individual is instructed to sequentially connect numbers within a circular pattern. In CTT-2, an additional challenge is introduced, requiring the numbers to be connected while alternating colors. The completion time of the test was measured, with a faster completion time resulting in a higher score. Standard scores, adjusted for age and educational level of the subjects, were obtained from the raw scores. The results from CTT tests were converted into standard scores and T-scores and the corresponding centile norms, from which a clinical interpretation of performance was obtained. The paper presented standardized TEN values. The higher the TEN score, the better the outcome [23].

Second, we used the Wisconsin test to assess executive functions. With it, the ability to reason abstractly and the ability to change cognitive strategies in response to changing environmental conditions can be assessed. The number of attempts needed to discern the sorting criteria was recorded, with fewer attempts leading to a higher score [24].

Metabolic Age

Body composition analysis was performed using a Tanita BC-601 Body Composition Analyzer (Tanita Europe B.V., Amsterdam, The Netherlands) at the surgery department and private preventive medicine center. Metabolic age is determined by comparing a person’s basal metabolism with the average metabolic rate of a person of the same chronological age using an algorithm built into the TANITA system [25].

### 2.4. Assessment of Metabolic Syndrome Components and Comorbidities

As part of routine testing, hemoglobin A1c (HbA1c) levels and lipid profiles were determined. During the assessment of comorbidities, parameters were used to assess the presence of prediabetes or diabetes and dyslipidemia. An HbA1c >= 5.7% was considered a parameter indicative of prediabetes, and an HbA1c >= 6.5% or a previous medical history of diabetes was considered indicative of diabetes. A value >= 3.4 mmol/l for the non-HDL parameter or the use of hypolipemic drugs was considered indicative of the presence of dyslipidemia. The patients’ medical history was checked for the presence of hypertension and other comorbidities.

### 2.5. Statistical Analysis

*p* = 0.05 was considered to indicate statistical significance. The choice of test (ANOVA vs. Kruskal–Wallis) depended on the conformity of the distribution of the variable in the subgroup to the normal distribution. Conformity to the normal distribution was assessed by analysis of histograms, kurtosis, skewness, and their standard errors. As there was a lack of conformity to the normal distribution in at least one subgroup for each of the quantitative variables analyzed, the nonparametric Kruskal–Wallis test was used for comparisons.

A linear regression method was used to assess the second endpoint. The main components of metabolic syndrome—hypertension, dyslipidemia, and prediabetes/diabetes—were selected as comorbidities.

Seven models were constructed in which parameters of inflammation (CRP or IL-6), telomere length, measures of cognitive function (CTT1-TEN, CTT2-TEN, and number of card-sorting test trials), and metabolic age parameters were used as the dependent variables. In models where the dependent variable was a parameter of inflammation, age, BMI, and the aforementioned components of metabolic syndrome were selected as predictors (independent variables). In the models with cognitive test scores, the same predictors were included as in the inflammation models, with the additional inclusion of CRP. CRP was chosen to standardize for possible additional somatic conditions not included in the model that could run with an increase in CRP and affect cognitive function (cancer, immune diseases, endocrine diseases, inflammation, etc.). Forced entry was chosen as the method for introducing predictors into the model. The following assumptions of the linear model were assessed: (1) linearity (analysis of scatter plots of the dependent variable and predictors) and (2) homoscedasticity, linearity, and normality of residuals (assessed by analyzing histograms of residuals and plots of standardized predicted values vs. standardized residuals). The data were analyzed for outliers and influential observations (by checking standardized residuals, Cook’s distance, the Mahalanobis distance, and ‘impact’ statistics (average leverage)). A variance inflation factor (VIF) was calculated for each model, where values > 10 indicate significant collinearity.

## 3. Results

### 3.1. Characteristics of the Group

No differences regarding age or sex were observed between the groups. The healthy controls had higher education levels than the patients with obesity. The two obesity subtypes did not differ in terms of obesity duration or BMI (Table 1).

### 3.2. The Impact of Obesity-Related Comorbidities on Premature Aging

Differences in chronological age, inflammatory markers (CRP, IL-6), telomere length, cognitive function (CTT-1 TEN, CTT-2 TEN, number of trials in Wisconsin Card Sorting Test), and metabolic age between a group of patients with obesity and comorbidity, obesity without comorbidity and healthy subjects were tested.

**Figure 1:**
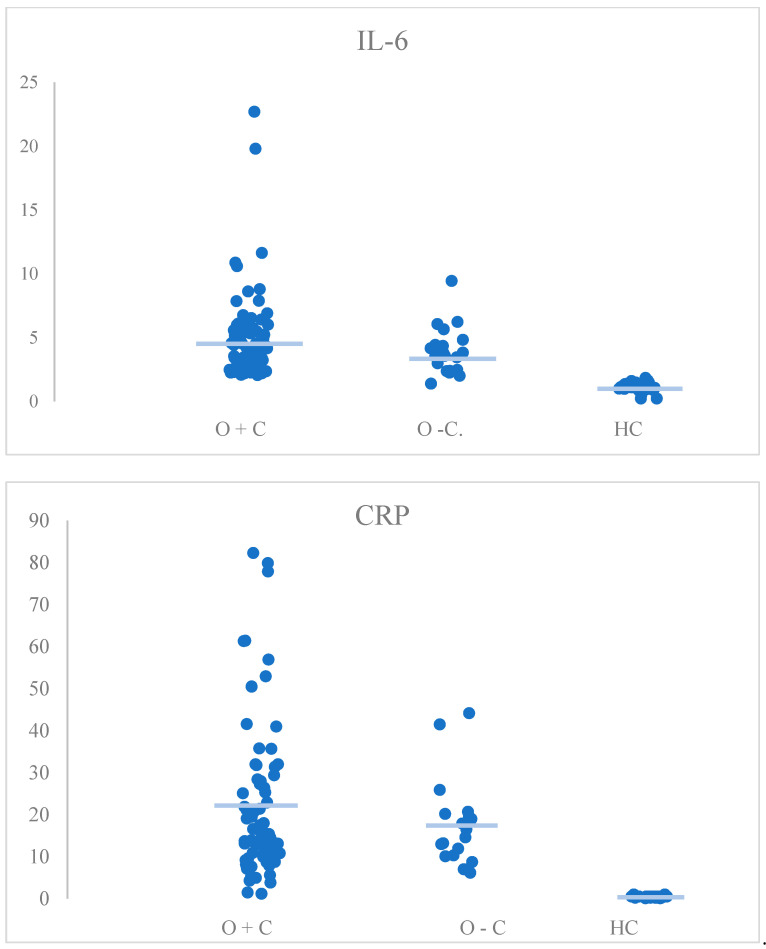

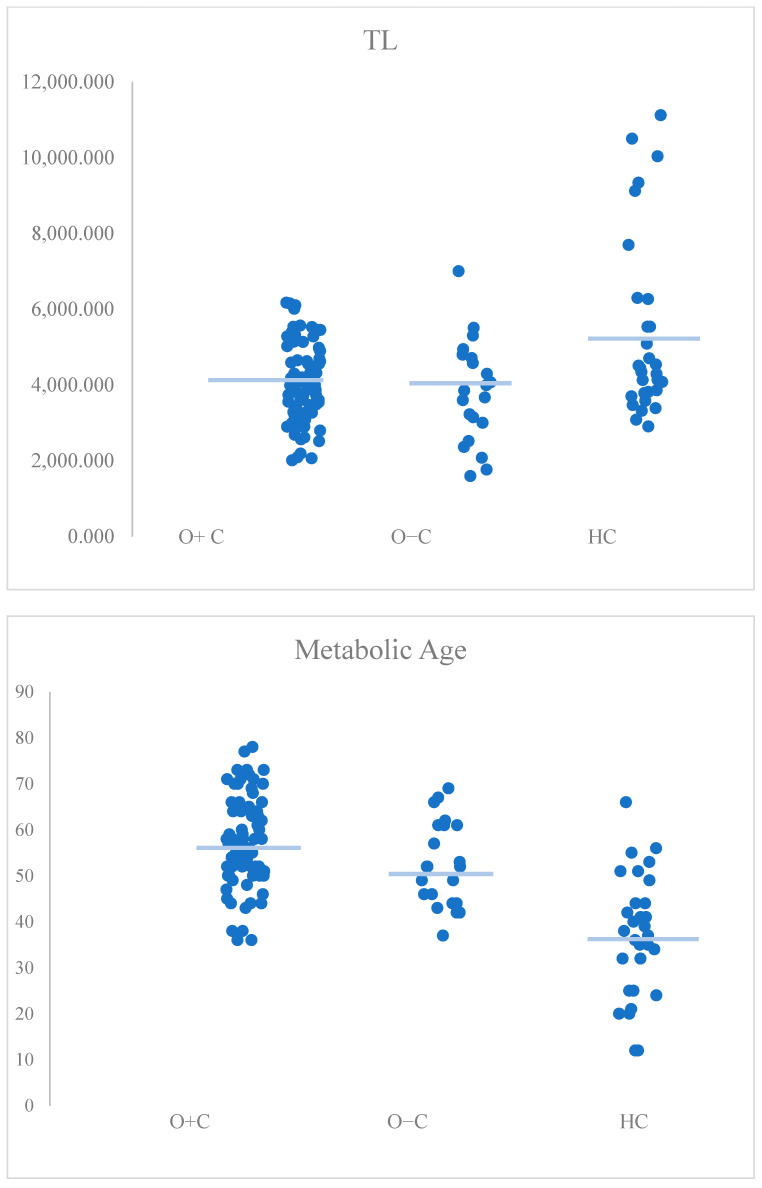

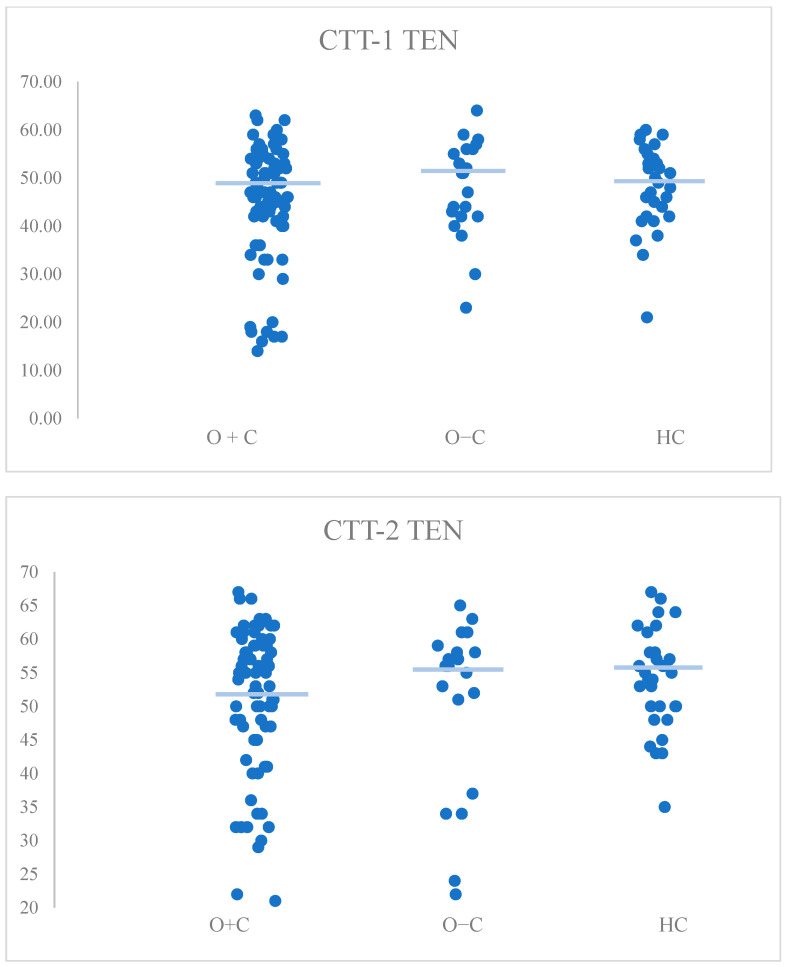

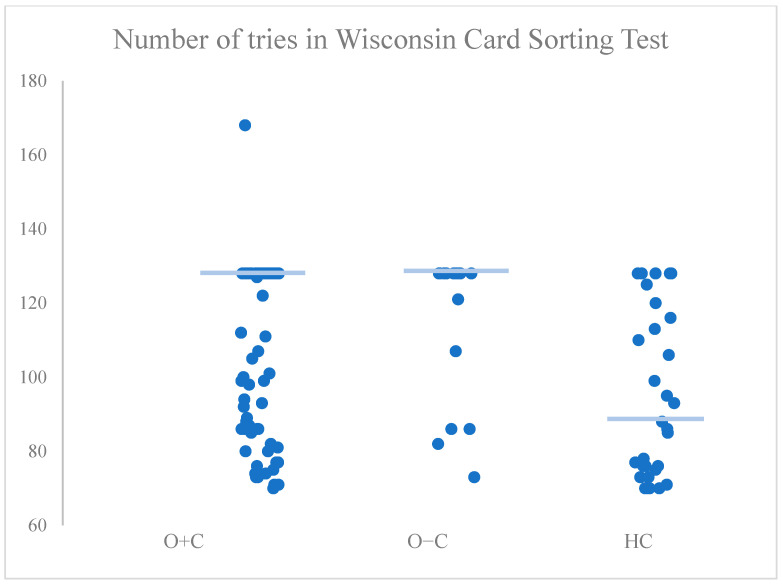
Dot plots representing the differences in levels of biological age markers between O+C, O−C, and HC. CRP—C-reactive protein, CTT-1 TEN—TEN results from the Color Trails Test, CTT-2 TEN—TEN results from the Color Trails Test 2, HC—healthy controls, IL-6—interleukin-6, O+C—patients with obesity and comorbidities, O−C—patients without comorbidities, TL—telomere length, blue line—median. The figure was created using Microsoft Excel version 16.66.1 (22101101).

The Kruskal–Wallis nonparametric test was used to assess the statistical significance of the differences presented (Table 2), (Figure 1).

Statistically significant differences in CRP levels were detected between the group with obesity and comorbidities (M = 15.25, IQR = 17.5) and the healthy group (M = 0.5, IQR = 0.3) (*p* < 0.001) and between the group with obesity without comorbidities (M = 15.6, IQR = 10.1) and the healthy group (M = 0.5, IQR = 0.3) (*p* < 0.001). Both groups with obesity had higher CRP levels than the control group. Comorbidities did not affect CRP levels among patients with obesity (*p* > 0.999).

Additionally, in terms of the IL-6 parameter, higher IL-6 levels were detected between the two groups of patients who were obese and the healthy group (M = 3.62, IQR = 2.9 vs. M = 1.15, IQR = 0.23), (*p* < 0.001) and (M = 3.81, IQR = 2.2 vs. M = 1.15, IQR = 0.23), *p* < 0.001), but no differences were detected between the subgroups of patients with obesity with and without comorbidities (*p* > 0.999).

### 3.3. Telomere Length

Patients with obesity had significantly shorter telomeres than healthy individuals (M = 3987, IQR = 1456 vs. M = 4392, IQR = 2510) (*p* = 0.022). The same difference was observed between patients with obesity without comorbidities and healthy individuals (M = 3851, IQR 1996 vs. M = 4392, IQR = 2510) (*p* = 0.043). No effect of comorbidities such as obesity on telomere length was observed. (*p* > 0.0999).

### 3.4. Cognitive Function

In terms of the CTT test, neither the CTT-1 (M = 47, IQR = 12 vs. M = 51, IQR = 14 vs. M = 49, IQR = 12) (*p* > 0.05) nor the CTT-2 (M = 51, IQR = 19 vs. M = 56, IQR = 23 vs. M = 55, IQR = 9) showed statistically significant differences between any of the groups (*p* > 0.999).

In terms of executive function, the Wisconsin Card Sorting Test showed a significantly worse outcome in patients with obesity with comorbidities than in healthy patients (M = 127, IQR = 42 vs. M = 87, IQR = 42), (*p* = 0.004) and patients with obesity without comorbidities than in healthy patients (M = 128, IQR = 17 vs. M = 87, IQR = 42), *p* = 0.001. Patients with obesity with or without comorbidities did not differ in the number of attempts to complete the test (*p* = 0.401)

### 3.5. Metabolic Age

Both groups of patients with obesity and without obesity presented significantly greater metabolic age (M = 57, IQR = 13 vs. M = 38, IQR = 20), (*p* < 0.001), (M = 52, IQR = 17 vs. M = 38, IQR = 20), (*p* = 0.001), but comorbidities did not differ between subgroups of patients with obesity (*p* > 0.999).

### 3.6. Effects of Individual Obesity-Related Diseases, Including Prediabetes/Diabetes, Hypertension, and Atherogenic Dyslipidemia, on Biological Age Markers

Linear regression was used to assess the potential relationships between BMI, comorbidities and inflammation, telomere length, cognitive function, or metabolic age. The main components of metabolic syndrome (prediabetes/diabetes, hypertension, atherogenic dyslipidemia) were selected as comorbidities.

Seven models with inflammatory parameters (CRP or IL-6), telomere length, measures of cognitive function (CTT1-TEN, CTT2-TEN, and the number of trials in the Wisconsin Card Sorting Test), and metabolic age as the dependent variables were built.

The detailed results of the regression model are described in the Online Supplementary Materials.

The model for CRP, CTT-1, and metabolic age was statistically significant.

The presence of prediabetes, diabetes, hypertension, or atherogenic dyslipidemia in obese individuals was not shown to significantly affect inflammatory parameters (CRP, IL-6). Only BMI had a significant effect on CRP levels.

The presence of prediabetes, diabetes, hypertension, or dyslipidemia has not been shown to affect telomere length.

In terms of cognitive functioning, a significant effect of the presence of hypertension in obese individuals on the deterioration of the CTT-1 TEN score according to the color-linked test was observed. Other components of metabolic syndrome that cooccur with obesity were not shown to significantly affect the deterioration of cognitive function. However, the effect of CRP on the deterioration of cognitive function was observed.

Additionally, no significant correlation was found between components of metabolic syndrome and metabolic age in patients with obesity. The analysis revealed that the sole determinant influencing the metabolic age parameter was the chronological age of the subjects under study.

## 4. Discussion

This is one of the few studies investigating the link between obesity-related comorbidities and aging in several aspects by indicators of inflammatory parameters, telomere length, cognitive function, and metabolic age.

An increasing number of studies are attempting to understand aging in individuals with obesity and highlight that obesity and diabetes contribute to accelerated aging, leading to the premature onset of the senescent state and contributing to the emergence of age-related diseases [26,27]. However, the role of comorbidities in obesity-induced aging is unclear. Some research indicates that accumulating metabolic diseases (hypertension, dyslipidemia, diabetes type 2) increase aging rates and cardiovascular incidents (Park et al.) [28]. Comorbidity is associated with increased levels of inflammatory markers [29,30,31], and it has been repeatedly demonstrated that the simultaneous presence of several diseases influences the premature death of patients hospitalized for various causes [32,33,34].

Our results, however, suggest that the influence of obesity on premature aging remains a robust factor independent of associated comorbidities.

We detected significant differences in biological age markers (CRP, IL-6, telomere length, metabolic age) between individuals with obesity and healthy controls, but no differences were detected between patients with and without comorbidities. In terms of cognitive function, both groups with obesity showed significant impairments in executive function, abstract reasoning, and adaptability compared to healthy controls, with no differences between the groups with and without comorbidities.

By examining the impact of metabolic syndrome components on premature aging, we found that hypertension in individuals with obesity may impair cognitive function. Additionally, elevated inflammation levels in obese individuals appear to affect cognitive abilities.

In a study by Diego T. Brunelli et al., similar conclusions were drawn, emphasizing the dominant role of obesity, regardless of associated disease, in increased gene expression of markers associated with inflammation and immunosenescence in circulating leukocytes of patients with obesity, with minimal additional impact from the presence of type 2 diabetes [35]. Wang et al. showed that obesity induces accelerated aging of T cells, leading to tumor progression, which is driven, at least in part, by leptin signaling [36]. Our findings also demonstrated that the significant increase in CRP parameters was attributed solely to BMI rather than comorbidities.

Obesity is linked to shorter telomeres, and diseases such as type 2 diabetes and cardiovascular disease further emphasize this effect [37,38]. Our study demonstrated that obesity alone influences shorter telomeres, regardless of other comorbidities, including diabetes, hypertension, or dyslipidemia. Similarly, Grun et al. reported that individuals with obesity, irrespective of comorbidities, had shorter telomeres, greater levels of negative regulators of the shelterin complex, increased lipid peroxidation, and elevated oxidized protein levels [39].

Comorbidities, as evidenced by studies such as Kim et al., are correlated with cognitive decline [31]. O.A. Trubinkov et al. reported that patients with diabetes and poorer metabolic profiles performed worse on neuropsychological tests after coronary artery bypass graft surgery than those without diabetes and who had healthier metabolic profiles [40]. On the other hand, in a study published by Anna M. Herghelegiu et al., selected metabolic parameters (fasting glucose, total cholesterol, LDL cholesterol, and triglyceride levels) did not correlate significantly with cognitive function [41]. In our study, we found that individuals with obesity, regardless of comorbidities, performed worse in cognitive tests, particularly in executive functions, and further analysis suggested that hypertension or inflammation accumulation in individuals with obesity might contribute to cognitive decline. Endothelial dysfunction, carotid stiffness, intima-media thickness, and increased inflammation of the microvasculature in individuals with hypertension might play a role in cognitive impairment in adults with obesity [42,43].

Our findings align with those of Gatto et al. [44], who identified hypertension as the only metabolic syndrome risk factor independently associated with lower cognitive function in verbal learning, semantic memory, and global cognition. Cavalieri et al. reported that metabolic syndrome worsens memory and executive function, particularly in men with high CRP levels [45]. The association between hypertension, CRP, and cognitive impairment in individuals with obesity indicates a greater risk of cerebral vascular damage and neurodegeneration in those with generalized atherosclerosis. Elevated inflammatory markers are linked to increased dementia risk, and obesity in youth is associated with dementia after age 65 [46,47].

Based on our findings, it is crucial to prioritize the treatment of obesity itself, independent of managing comorbidity parameters, to mitigate the risk of premature aging. Future research is necessary to determine the specific stage of obesity at which premature aging becomes evident.

### Strength and Limits

The relationships among obesity, comorbidities, and aging remain unclear due to inconsistencies in age markers, methodological variations, demographic differences, and challenges in isolating specific effects of obesity-related conditions. Our study also has several limitations, including a small sample size due to funding constraints and an uneven distribution of participants, with few individuals with obesity without comorbidities. This reflects the rarity of severe obesity without comorbidities.

## 5. Conclusions

In conclusion, severe obesity, regardless of the associated disease, induces increased premature aging. Additionally, the presence of hypertension in individuals with severe obesity and increased inflammatory levels might influence the worsening of cognitive function.

## Figures and Tables

**Table 1:** Characteristics of the groups.

Parameter	O+C	O−C	HC
Total, n	78	21	30
Mean age, years (M ± SD)	43.33 ± 10.49	38.4 ± 8.51	44.5 ± 8.55
Sex (female), n (%)	27 (34.6)	7 (33.3)	10 (33.3)
Education			
primary, n (%)	1 (1.3) *^&^	0 (0)	0 (0)
vocational, n (%)	3 (3.84) *^&^	3 (14.3) ^&^	0 (0)
secondary, n (%)	35 (44.88) *^&^	4 (19.0) ^&^	0 (0)
higher education, n (%)	39 (50) ^&^	14 (66.7) ^&^	30 (100)
Prediabetes/diabetes, n (%)	47 (60.3)	-	-
Hypertension, n (%)	36 (46.2)	-	-
Atherosclerosis, n (%)	34 (43.6)	-	-
Arthritis, n (%)	5 (6.4)	-	-
Psoriasis, n (%)	5 (6.4)	-	-
PCOS, n (%)	2 (2.6)	-	-
Nonalcoholic fatty liver disease, n (%)	3 (3.8)	-	-
Hypothyroidism, n (%)	13 (16.7)	-	-
Graves-basedov disease, n (%)	3 (3.00)	-	-
Obstructive sleep apnea, n (%)	2 (2.6)	-	-
Asthma, n (%)	5 (6.4)	-	-
COPD, n (%)	1 (1.3)	-	-
Duration of obesity, years (M ± SD)	20.31 ± 11.2	20.31 ± 10.02	-
BMI (kg/m^2^), (M ± SD)	44.98 ± 6.06 ^&^	44.34 ± 6.3 ^&^	23.06± 3.13
Muscle mass, kg (M ± SD)	65.45 ± 14.13 ^&^	64.83 ± 11.13 ^&^	50.88 ± 12.21
Percentage of body fat, % (M ± SD)	46.15 ± 6.83 ^&^	45.6± 7.24 ^&^	24.3 ± 7.97
Visceral fat index (n), M ± SD	19 ± 8 ^&^	18 ± 7 ^&^	5 ± 3
Basal metabolic rate (kcal)	2201 ± 328 ^&^	2179 ± 481 ^&^	1578 ± 362

BMI, body mass index. * Significantly different from O−C, ^&^ Significantly different from HC; *p* ≤ 0.05.

**Table 2:** The differences in chronological age markers between O+C, O−C, and HC according to the Kruskal–Wallis test.

	Kruskal–Wallis Test Parameters	Pairwise Comparisons ^a^
Chronological age	H(2) = 5.88, *p* = 0.053	HC vs. O+C: *p* > 0.999 HC vs. O−C: *p* = 0.057 O+C vs. O−C: *p* = 0.124
CRP	H(2) = 68.11, *p* < 0.001	HC vs. O+C: *p* < 0.001 * HC vs. O−C: *p* < 0.001 * O+C vs. O−C: *p* > 0.999
IL-6	H(2) = 68.25, *p* < 0.001	HC vs. O+C: *p* < 0.001 * HC vs. O−C: *p* < 0.001 * O+C vs. O−C: *p* > 0.999
Telomere length	H(2) = 8.28, *p* = 0.016	HC vs. O+C: *p* = 0.028 * HC vs. O−C: *p* = 0.043 * O+C vs. O−C: *p* > 0.999
CTT1-TEN	H(2) = 1.08, *p* = 0.584	HC vs. O+C: *p* > 0.999 HC vs. O−C: *p* > 0.999 O+C vs. O−C: *p* > 0.999
CTT2-TEN	H(2) = 2.7, *p* = 0.260	HC vs. O+C: *p* = 0.327 HC vs. O−C: *p* > 0.999 O+C vs. O−C: *p* > 0.999
Number of trials	H(2) = 15.52, *p* < 0.001	HC vs. O+C: *p* = 0.005 * HC vs. O−C: *p* = 0.001 * O+C vs. O−C: *p* = 0.369
Metabolic age	H(2) = 42.05, *p* < 0.001	HC vs. O+C: *p* < 0.001 * HC vs. O−C: *p* = 0.001 * O+C vs. O−C: *p* = 0.525

^a^—Adjusted with Bonferroni correction, CRP—C-reactive protein, CTT1-TEN—TEN results from the Color Trails Test, CTT2-TEN—TEN results from the Color Trails Test 2, HC—healthy controls, IL-6—interleukin-6, O+C—patients with obesity and comorbidities, O−C—patients without comorbidities, number of trials—number of tries in the Wisconsin Card Sorting Test, * *p* < 0.05.

## Data Availability

The data that support the findings of this study are not openly available and are available from the corresponding author upon reasonable request. The administrator of the database is the Jagiellonian University Medical College in Krakow.

## Supplementary Materials

The following supporting information can be downloaded at https://www.mdpi.com/article/10.3390/medicina61020293/s1. Table S1: Linear regression models on associations between BMI, comorbidities (prediabetes/diabetes, hypertension, and atherogenic dyslipidemia), and biological age markers: inflammatory markers (CRP and IL-6), telomere length, cognitive function assessment: TEN results of Color Trails Test (CTT-1 and CTT-2 TEN), number of trials in Wisconsin Card Sorting Test, and metabolic age parameter.
