# Supplementary material for: What Is the Impact of Obesity-Related Comorbidities on the Risk of Premature Aging in Patients with Severe Obesity?: A Cross-Sectional Study
Source: Medicina (Kaunas). 2025 Feb 8;61(2):293. doi: 10.3390/medicina61020293 (PMC11857414; doi:10.3390/medicina61020293)
Supplement: Supplementary file 1 [file medicina-61-00293-s001.zip › medicina-3398937-supplementary.pdf]

Supplementary Materials

Table S1. Linear regression models on associations between BMI, comorbidities (prediabetes/diabetes, hypertension, atherogenic dyslipidemia) and biological age markers: inflammatory markers (CRP, IL-6), telomere length, cognitive function assessment: TEN results of Color Trails Test (CTT-1, CTT-2 TEN), number of trials in Wisconsin Card Sorting Test, metabolic age parameter.

Model for CRP as a dependent variable. B- unstandardized coefficient; CI – confidence interval; SE – standard error; β – standardized coefficient. R^2 – Coefficient of Determination.					
	b	95% CI for b	SE	β	p
Age	0.098	-0.277; 0.473	0.189	0.060	0.604
Pre-diabetes/diabetes	3.090	-4.224; 10.404	3.678	0.092	0.403
Hypertension	1.905	-5.355; 9.164	3.651	0.055	0.603
Atherogenic dyslipidemia	-6.039	-13.178; 1.100	3.591	-0.173	0.096
BMI	0.982	0.459; 1.504	0.263	0.372	<0.001
R^2=0.17, p_{model}=0.007					

Model for IL-6 as a dependent variable. B- unstandardized coefficient; CI – confidence interval; SE – standard error; β – standardized coefficient. R2 – Coefficient of Determination.					
	b	95% CI for b	SE	β	p
Age	-0.052	-0.126; 0.022	0.037	-0.168	0.163
Pre-diabetes/diabetes	1.377	-0.049; 2.802	0.718	0.216	0.058
Hypertension	1.044	-0.374; 2.462	0.714	0.158	0.147
Atherogenic dyslipidemia	-0.569	-1.968; 0.831	0.705	-0.085	0.422
BMI	0.001	-0.101; 0.103	0.051	0.002	0.985
R^2=0.06, p_{model}=0.317					

Model for telomere length as a dependent variable. B- unstandardized coefficient; CI – confidence interval; SE – standard error; β – standardized coefficient. R2 – Coefficient of Determination.					
	B	95% CI for b	SE	β	p
Age	5.351	-20,465; 31,168	12.980	0.050	0.681
Pre-diabetes/diabetes	310.543	-198,219; 819,306	255.793	0.140	0.228
Hypertension	60.851	-440,939; 562,641	252.288	0.027	0.810
Atherogenic dyslipidemia	-493.709	-995,973; 8,556	252.526	-0.217	0.054
BMI	41.142	2,389; 79,895	19.484	0.239	0.038
CRP	-3.369	-18,225; 11,488	7.469	-0.52	0.653
R^2=0.10, p_{model}=0,180					

Model for CTT1-TEN as a dependent variable. B- unstandardized coefficient; CI – confidence interval; SE – standard error; β – standardized coefficient. R2 – Coefficient of Determination.					
	b	95% CI for b	SE	β	p
Age	-0.013	-0.272; 0.246	0.130	-0.012	0.921
Pre-diabetes/diabetes	-2.726	-7.790; 2.338	2.546	-0.118	0.287
Hypertension	-6.907	-11.921; -1.894	2.521	-0.290	0.008
Atherogenic dyslipidemia	0.967	-4.037; 5.971	2.516	0.041	0.702
BMI	-0.050	-0.439; 0.338	0.195	-0.028	0.797
CRP	-0.150	-0.299; -0.002	0.075	-0.220	0.048
R^2=0.17, p_{model}=0.036					

Model for CTT2-TEN as a dependent variable. B- unstandardized coefficient; CI – confidence interval; SE – standard error; β – standardized coefficient. R2 – Coefficient of Determination.					
	b	95% CI for b	SE	β	p
Age	0.041	-0.245; 0.368	0.149	0.032	0.786
Pre-diabetes/diabetes	-3.087	-8.666; 3.312	2.920	-0.120	0.293
Hypertension	-3.831	-9.441; 2.425	2.891	-0.145	0.189
Atherogenic dyslipidemia	-0.110	-5.709; 6.134	2.886	-0.004	0.970
BMI	0.139	-0.336; 0.585	0.224	0.069	0.536
CRP	-0.215	-0.390; -0.037	0.086	-0.282	0.014
R^2=0.10, p_{model}=0.183					

Model for the number of trials in card sorting test. B- unstandardized coefficient; CI – confidence interval ; SE – standard error; β – standardized coefficient. R2 – Coefficient of Determination.					
	b	95% CI for b	SE	β	p
Age	0.872	0.346; 1.397	0.264	0.398	0.001
Pre-diabetes/diabetes	-3.118	-13.405; 7.168	5.171	-0.069	0.548
Hypertension	-5.260	-15.531; 5.010	5.163	-0.112	0.311
Atherogenic dyslipidemia	-0.860	-11.078; 9.359	5.137	-0.018	0.867
BMI	0.064	-0.728; 0.856	0.398	0.018	0.873
CRP	0.087	-0.213; 0.387	0.151	0.065	0.566
R^2=0.12, p_{model}=0.086					

Model for the metabolic age parameter. B- unstandardized coefficient; CI – confidence interval ; SE – standard error; β – standardized coefficient. R2 – Coefficient of Determination.					
	b	95% CI for b	SE	β	p
Age	0.906	0.793; 1.019	0.057	0.920	<0.001
Pre-diabetes/diabetes	-1.018	-3.199; 1.163	1.096	-0.051	0.356
Hypertension	-0.559	-2.715; 1.596	1.083	-0.027	0.607
Atherogenic dyslipidemia	0.135	-2,049; 2,319	1.098	0.007	0,903
BMI	0.161	-0.008; 0.331	0.085	0.103	0,062
CRP	-0.008	-0.072;0.056	0.032	-0.014	0,798
R^2=0.80, p_{model}<0,001					
B- unstandardized coefficient; CI – confidence interval ; SE – standard error; β – standardized coefficient. R2 – Coefficient of Determination.					
